# Tomato Dynamin Related Protein 2A Associates With LeEIX2 and Enhances PRR Mediated Defense by Modulating Receptor Trafficking

**DOI:** 10.3389/fpls.2019.00936

**Published:** 2019-07-19

**Authors:** Lorena Pizarro, Meirav Leibman-Markus, Silvia Schuster, Maya Bar, Adi Avni

**Affiliations:** ^1^School of Plant Sciences and Food Security, Tel Aviv University, Tel Aviv, Israel; ^2^Department of Plant Pathology and Weed Research, Agricultural Research Organization, Volcani Center, Rishon, Israel

**Keywords:** LeEIX2, EIX, tomato, dynamin related protein, DRP2A, endomembrane trafficking, defense responses

## Abstract

The endocytic trafficking pathway is employed by the plant to regulate immune responses, and is often targeted by pathogen effectors to promote virulence. The model system of the tomato receptor-like protein (RLP) LeEIX2 and its ligand, the elicitor EIX, employs endocytosis to transmit receptor-mediated signals, with some of the signaling events occurring directly from endosomal compartments. Here, to explore the trafficking mechanism of LeEIX2-mediated immune signaling, we used a proteomic approach to identify LeEIX2-associating proteins. We report the identification of SlDRP2A, a dynamin related protein, as an associating partner for LeEIX2. SlDRP2A localizes at the plasma membrane. Overexpression of SlDRP2A increases the sub-population of LeEIX2 in VHAa1 endosomes, and enhances LeEIX2- and FLS2-mediated defense. The effect of SlDRP2A on induction of plant immunity highlights the importance of endomembrane components and endocytosis in signal propagation during plant immune responses.

## Introduction

The perception of environmental signals and the ability to respond accordingly are essential for organism survival. Plants encounter a vast plethora of potential pathogens, but seldom do these microbial overtures lead to disease. Nevertheless, pathogens still cause significant crop losses. Plant defense mechanisms depend on the capacity of each individual cell to initiate immune responses (Couto and Zipfel, 2016). Plant immunity can be triggered by elicitors such as microbe-associated molecular patterns (MAMPs) (Henry et al., 2013). MAMPs have been isolated from a variety of pathogenic and non-pathogenic micro-organisms. This type of immunity is termed pattern-triggered immunity (PTI). In the past decade, MAMP recognition and PTI have been intensively studied (Macho et al., 2014; Bigeard et al., 2015). PTI is sufficient to ward off most microbes. Pathogens must evade or actively suppress this first layer of immunity in order to successfully cause disease (Dodds and Rathjen, 2010).

Plants sense MAMPs through cell surface pattern recognition receptors (PRRs) (Boutrot and Zipfel, 2017). PRRs directly bind their ligands, resulting in rapid changes in the cell (Macho et al., 2014). PRRs are either receptor-like kinases (RLKs), or receptor-like proteins (RLPs). RLKs possess a single transmembrane domain, an intracellular kinase domain for signal transduction (Boutrot and Zipfel, 2017), and some also contain an extracellular leucine-rich repeat (eLRR) domain potentially involved in ligand binding. RLPs share the same basic structure, but lack a potential intracellular signaling domain. RLPs are assumed to rely on other components to initiate a signaling cascade, possibly forming a hetero receptor complex with RLKs or with receptor-like cytoplasmic kinases to transduce a signal to downstream target proteins (Tor et al., 2009; Gust and Felix, 2014).

In tomato (*Solanum lycopersicum*) the RLP LeEIX2 recognizes and responds to the fungal MAMP-EIX (ethylene-inducing xylanase) (Ron and Avni, 2004). EIX induces ethylene production, extensive electrolyte leakage, and hypersensitive response (HR) (Bailey et al., 1990, 1991; Sharon et al., 1992). EIX was shown to specifically bind to the plasma membrane (PM) of responsive cultivars of tomato and tobacco (Hanania et al., 1997; Sharfman et al., 2011). The binding of EIX to LeEIX2 induces receptor-mediated endocytosis, thus allowing the receptor to interact with cytoplasmic proteins and generate a signal to induce defense responses (Ron and Avni, 2004). Inhibition of endosome formation reduces EIX mediated responses, while arresting post-endocytosis traffic increases these responses, indicating signals for propagation of defense responses are emanating from endosomes (Sharfman et al., 2011).

In recent years, the role of receptor mediated endocytosis (RME) in plant immunity has been studied for several plant PRRs, such as FLS2, EFR, PEPR, LYK5, CERK1, and LeEIX2 (Bar and Avni, 2009; Bar et al., 2010; Ben Khaled et al., 2015; Toruno et al., 2016; Valencia et al., 2016; Gu et al., 2017; Yun and Kwon, 2017).

Receptor-mediated endocytosis has long been recognized as a mechanism of signaling initiation and termination via degradation of activated receptor complexes after their internalization from the cell surface (Claus et al., 2018). In metazoans, a large body of evidence supports the notion that signaling is not restricted to the PM (Gould and Lippincott-Schwartz, 2009; Sorkin and von Zastrow, 2009). Evidence of endosomal signaling following receptor endocytosis in plants was reported for the BRI1, FLS2, PEPR1, and LeEIX2 receptors (Geldner et al., 2007; Sharfman et al., 2011; Smith et al., 2014; Mbengue et al., 2016; Ortiz-Morea et al., 2016).

Trafficking regulators are subject to transcriptional and/or posttranslational regulation, having significant activity changes in response to multiple forms of immune signals (Valencia et al., 2016). Manipulations of critical trafficking regulators have been shown to be key for successful pathogen infection and/or mounting of plant defenses in many cases (Ben Khaled et al., 2015; Toruno et al., 2016; Pizarro et al., 2018).

Dynamin related proteins (DRPs) are mechanochemical GTPase’s involved in the scission of endocytic clathrin coated vesicles (CCV) from membranes during clathrin-mediated endocytosis (CME) (Heymann and Hinshaw, 2009; McNew et al., 2013). DRPs play a role in resistance against bacterial and fungal pathogens in several cases (Tang et al., 2006; Smith et al., 2014; Chaparro-Garcia et al., 2015). DRP2s in particular have been shown to play a role in immunity: DRP2 is required for FLS2 receptor-mediated endocytosis after flg22 elicitation (Chaparro-Garcia et al., 2015). AtDRP2B affects PTI in response to both flg22 and *Pseudomonas syringe* (Smith et al., 2014). DRP2 also interacts with the effector – AvR3a, a suppressor of FLS2 internalization and PTI responses (Chaparro-Garcia et al., 2015). Here, we report the identification of tomato DRP2A as an associating partner for the RLP LeEIX2 and demonstrate its involvement in LeEIX2- and FLS2-mediated signaling, highlighting the importance of endomembrane components in signal propagation during plant immune responses.

## Materials and Methods

### Plant Materials and Growth Conditions

*Nicotiana tabacum* cv samsun NN, *Nicotiana benthamiana*, and *S. lycopersicum* cv M82 and IL7-5 were grown from seeds in soil (Green Mix; Even-Ari, Ashdod, Israel) in a growth chamber, under long day conditions (16 h:8 h, light:dark) at 24°C.

### Plasmid Construction

For overexpression assays, LeEIX2 cDNA C-terminally tagged with GFP was cloned into the SalI site of pBINPLUS (van Engelen et al., 1995) as described by Pizarro (Pizarro et al., 2018). SlDRP2A (Solyc11g039650) cDNA C-terminally tagged with GFP, mCherry or 2xHA was cloned into the *Sal*I *Xba*I sites of pBINPLUS (van Engelen et al., 1995) using the following primers: SlDRP2A forward primer 5′-AGGTCGACATGGAAGCAATC GAGGAATTG-3′ and SlDRP2A reverse primer 5′-CCTCTA GAAGATCTATAACCGGATCCAGAAG-3′ between the CAM35SΩ promoter containing the translation enhancer signal and the Nos terminator. Site-directed mutagenesis of lysine to alanine in residue 53 was generated using the full-length plasmid and sub-cloned as described above using the following primers: SlDRP2AK53A forward primer 5′-TGGTGCTGGTGCATCAGC TGTAC-3′ SlDRP2AK53A reverse primer 5′-GTGCCACCG ATAGCAACA-3′.

### Transient Expression by Agroinfiltration

Binary vector clones were introduced by electroporation into *Agrobacterium tumefaciens* strain GV3101. Agrobacterium cells were grown in LB medium containing 50 mg/L Kanamycin or 100 mg/L Spectinomycin, 40 mg/L Gentamycin and 100 mg/L Rifampicin overnight at 28^∘^C, diluted into VIR induction medium (50 mM MES pH 5.6, 0.5% (w/v) glucose, 1.7 mM NaH_2_PO_4_, 20 mM NH_4_Cl, 1.2 mM MgSO_4_, 2 mM KCl, 17 μM FeSO_4_, 70 μM CaCl_2_, and 200 μM acetosyringone) and grown for six additional hours until OD600 reached 0.4–0.6. Suspensions containing single or mixed Agrobacterium cultures were diluted to a final OD_600_ of 0.15–0.2 in VIR induction medium. Cultures were infiltrated with a needleless syringe into leaves of *N. tabacum* cv samsun NN or *N. benthamiana*. Leaves were harvested 40 h after injection for ethylene biosynthesis and ROS assays, co-immunoprecipitation or confocal microscopy analysis.

### LeEIX2 Associated Protein Identification by Immunopurification, Followed by Tryptic Digest and Mass Spectrometry

Immunopurification from the transgenic tomato line expressing LeEIX2-GFP were performed as described by Leibman-Markus et al. (2018). Plant leaves were harvested for immunopurification and proteins were extracted using detergent free extraction buffer (EB) and protease inhibitor tablet (Roche, Germany). Pellets were ground with EB containing 0.5% Triton X-100. The supernatant was diluted with detergent free EB in order to obtain a final concentration of 0.2% Triton^TM^ x-100 before adding GFP-TrapA beads (Chromotek, Planegg-Martinsried, Germany). Beads were incubated for 4 h and then washed five times with detergent free EB. Tryptic on-bead digestion was performed. Peptides were subjected to mass spectrometry as previously described (Liu et al., 2011). Mass spectra were detected on a Q Exactive mass spectrometer (Thermo Fisher Scientific). Raw mass spectrometry data were searched with X!Tandem version Sledgehammer against the tomato proteome ITAG2.3 (SolGenomics^1^) and imported into Scaffold 4.8.3 (Proteome Software) as described by Yadeta et al. (2017). Mass spectrometry was performed at the UC Davis proteomics core.

### Co-immunoprecipitation

Co-immunoprecipitation assays were performed as described by Leibman-Markus et al. (2017): *N. benthamiana* leaves transiently coexpressing LeEIX2-GFP or LeEIX2 and SlDRP2A-HA were harvested 40 h after infiltration. Leaf petioles were immersed in EIX 300 μg/ml (or water as mock) for 7 min, and then transferred to water for an additional 7 min. Five hundred mg leaf tissue was used for co-immunoprecipitation, with 13 μl GFP-TrapA beads (Chromotek, Planegg-Martinsried, Germany). Beads were incubated for 4 h and then washed five times with detergent free EB. Immune-precipitated (IP) and input samples were run in SDS-PAGE, blotted onto nitrocellulose membranes and incubated with antibodies as required: rat anti-GFP (Chromotek, Planegg-Martinsried, Germany) and mouse anti-HA (BioLegend, CA, United States).

### ROS Measurement

ROS burst was measured as previously described by Leibman-Markus et al. (2017). Leaf disks (0.5 cm diameter) were taken from either transiently expressing tobacco plants 40 h post-infection or stable transgenic tomato lines. Disks were floated in 250 μl ddH_2_O in a white 96-well plate (SPL Life Sciences, Korea) for 4–6 h at room temperature. After incubation, the water was completely removed and ROS measurement reaction containing EIX 1 μg/ml or DMSO as mock was added and light emission was immediately measured using a micro-plate luminometer (Turnerbiosystems Veritas, Sunnyvale, CA, United States).

### Ethylene Measurement

Ethylene biosynthesis was measured as previously described by Leibman-Markus et al. (2017). Leaf disks (0.9 cm diameter) were taken from transiently expressing tobacco plants 40 h post-infection. Five disks were sealed in each 10 ml flask containing 1ml assay medium (with or without 1 μg/ml EIX) and incubated with shaking for 4 h at room temperature. Ethylene production was measured by Gas chromatography (Varian 3350, Varian, CA, United States).

### Hypersensitive Response Measurement

*Nicotiana tabacum* leaves were transiently transformed with agrobacteria harboring free GFP (control), SlDRP2A-GFP or SlDRP2AK53A-GFP. Twenty-four hours post-agrobacteria infiltration, leaves were infiltrated into the abaxial side with a needless syringe, with 1 μg/ml EIX. Twenty-four hours after EIX elicitation, leaves were photographed and the images analyzed using ImageJ for area quantification of HR development measurements. The infiltrated and HR areas were measured, HR was calculated as percentage of infiltrated area.

### Bioinformatic Analyses

*Arabidopsis thaliana* DRP sequences were used to identify homologs in the Tomato Genome (ITAG release 2.40). The phylogenetic tree of *A. thaliana* and *S. lycopersicum* DRPs was built using multiple alignment (CLUSTAL_W) and maximum likelihood (Phylogeny.fr) methods, with bootstrap values based on 1,000 iterations (Dereeper et al., 2008). Protein domain prediction for SlDRP2A was performed using Conserved Domain search from National Center of Biotechnology Information website^2^ (Marchler-Bauer et al., 2017).

### Confocal Microscopy

Confocal microscopy images were acquired using a Zeiss LSM780 confocal microscope system with Objective C-Apochromat 40x/1.2 W Corr M27. Images were acquired using two tracks. Track 1 collected the chlorophyll fluorescence using an excitation laser wavelength of 633 nm (2% power) and an emission collection range of 652–721 nm. Track 2 used two different channels to collect GFP and dsRed or mCherry fluorescence using an excitation laser of 488 nm (5% power) and 561 nm (3% power), respectively. For GFP, the emission was collected in the range of 493–535 nm and for mCherry emission was collected in the range of 588–641. Images of 8 bits and 1024X1024 pixels were acquired using a pixel dwell time of 1.27, pixel averaging of 4 and pinhole of 1 airy unit. Z-stacks were acquired ensuring an overlap of 50% for each slice. For EIX treatment, 1 cm disks of tissue were incubated for 7 min with 1 μg/ml EIX or water, then the samples were mounted for imaging.

Image analysis was conducted with Fiji-ImageJ using the raw images (Schindelin et al., 2012). We used the Coloc2 tool for Colocalization analysis and the 3D Object counter tool for quantifying endosome numbers (Schindelin et al., 2012).

## Results

### Tomato DRP Associates With LeEIX2

**FIGURE 1:**
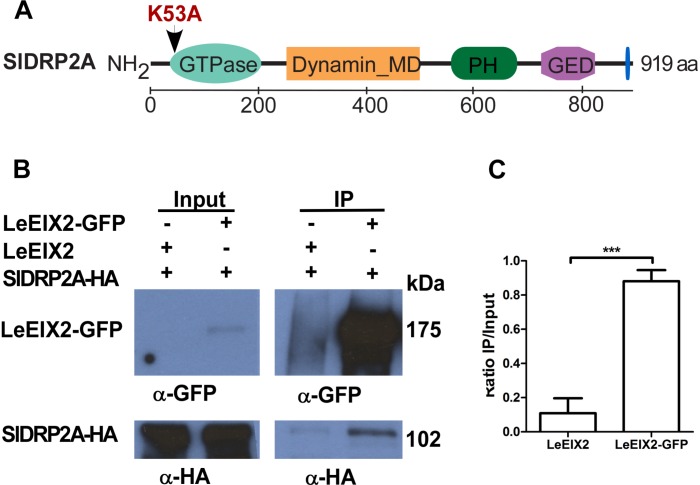
Co-immunoprecipitation of SlDRP2A and LeEIX2. **(A)** Schematic representation of SlDRP2A domain architecture. Domains were identified using NCBI conserved domains: GTPase 43–305 aa, Dynamin middle domain (MD) 259–492 aa, Pleckstrin Homology (PH) domain 579–701, GTPase effector domain (GED) 745–818 aa and Proline Rich Motif (PRM) 906–910 aa (Marchler-Bauer et al., 2017). Mutation in the GTP binding site at position 53 aa, generating the SlDRP2AK53A mutated version, is marked in red. **(B)** *N. benthamiana* was transiently co-transformed with LeEIX2-GFP or untagged LeEIX2 (control) and SlDRP2A-HA. Immunoprecipitation (IP) using GFP affinity beads was performed on TSM protein fractions. Input and IP samples were subjected to SDS-PAGE and immunoblot analyses with α-GFP and α-HA antibodies to detect LeEIX2-GFP and SlDRP2A-HA. **(C)** SlDRP2A-HA signal intensity was determined using FIJI-ImageJ. Ratio of co-IP intensity and input intensity was calculated for each sample. Error bars represent the average ± SD of three independent experiments. Asterisks indicate significant differences (one-way ANOVA, Tukey post-test, ^∗∗∗^*p* < 0.001).

In order to identify LeEIX2 associating proteins, we utilized proteomic techniques and a tomato line overexpressing LeEIX2 fused to eGFP (Leibman-Markus et al., 2018). Purified proteins were subjected to tryptic on-bead digestion, and tryptic peptides were analyzed by mass spectrometry. This analysis identified peptides matching a predicted protein encoded by tomato Solyc11g039650, a putative DRP (Supplementary Table S1). Bioinformatic analysis predicts that this locus encodes a homolog of DRP of type 2, and it is called hereinafter SlDRP2A (Figure 1A and Supplementary Figure S1). In order to further demonstrate the association between LeEIX2 and SlDRP2A, we performed CoIP. SlDRP2A-HA and LeEIX2-GFP were transiently coexpressed in *N. benthamiana* and LeEIX2-GFP was pulled down using GFP affinity beads. SlDRP2A-HA was successfully co-purified in the presence of LeEIX2-GFP indicating an association *in planta* and corroborating the proteomic analysis (Figures 1B,C).

SlDRP2A is a homolog of AtDRP2A and AtDRP2B, sharing 78 and 77% identity at protein level, respectively (Supplementary Figure S1). AtDRP2A and AtDRP2B belong to the DRP2 subfamily of *bonafide* dynamins (Hong et al., 2003; Backues et al., 2010; Chappie and Dyda, 2013; Fujimoto and Tsutsumi, 2014). Bioinformatic analysis revealed an additional tomato putative DRP clustering to the DRP2 – plant dynamin subfamily, designated XP_004230421 in NCBI (Supplementary Figure S1). We have designated this protein SlDRP2B. SlDRP2B possesses 75.2% identity and 86.5% similarity with SlDRP2A at protein level (Supplementary Figure S2). In the tomato genome, SlDRP2B is erroneously annotated as two separate, truncated, consecutive loci, designated Solyc01g103130 and Solyc01g103120. DRP2 subfamily proteins contain the three dynamin core domains; an N-terminal GTPase domain, a middle region harboring a coiled-coil domain, and a GTPase effector domain (GED; Figure 1A) (van der Bliek, 1999; Ramachandran et al., 2007). Additionally, DRP2s contain a Pleckstrin Homology (PH) domain involved in preferential targeting to phosphatidylinositol (4,5)-biphosphate lipids, which is enriched in the PM; and a Proline-Rich Motif (PRM) that binds phospholipids and proteins carrying the SH3 domain (Figure 1A) (Hong et al., 2003; Ramachandran et al., 2007; Simon et al., 2014).

### SlDRP2A Localizes at the Plasma Membrane and Partially to the EE/TGN

**FIGURE 2:**
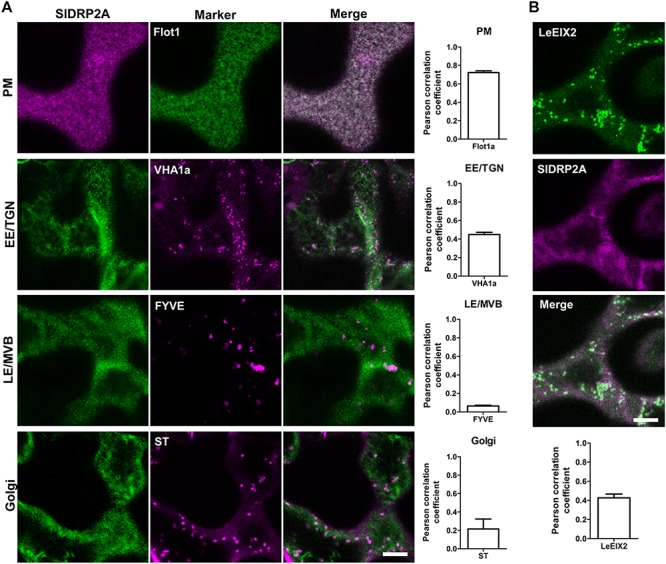
SlDRP2A subcellular localization. Confocal microscopy of *N. benthamiana* epidermal cells transiently expressing SlDRP2A–(GFP or mCherry), representative images as shown. **(A)** Different endomembrane compartment markers as indicated fused to GFP, dsRed or mCherry, coexpressed with SlDRP2A-(GFP or mCherry). Colocalization between SlDRP2A and the endomembrane markers was analyzed using Coloc2 from FIJI-ImageJ. Representative images and Pearson correlation coefficient are shown. Data represented as mean ± SEM. *N* ≥ 22 images. **(B)** Coexpression of LeEIX2-GFP and SlDRP2A-mCherry. Colocalization between SlDRP2A and LeEIX2 was determined using Coloc2 from FIJI-ImageJ. Scale bar = 10μm. For colocalization, ≥ 20 images were used, graph shows average ± SEM.

To further characterize SlDRP2A and determine its subcellular localization, we conducted live cell imaging, using fluorescently labeled SlDRP2A. Confocal images of *N. benthamiana* epidermal cells reveal that SlDRP2A colocalizes with the PM marker Flot1 (Li et al., 2012), with a Pearson correlation coefficient of 0.72 ± 0.025 (Figure 2A and Supplementary Figure S3A). SlDRP2A and Flot1 localize on discrete foci at the PM (Figure 2A and Supplementary Figure S3A). These type of foci have been previously described as nano-domains of several PM proteins such as Flot1, as well as the RLK proteins BRI, FLS2, and LYK3 among others (Wang et al., 2015; Bucherl et al., 2017; Ott, 2017; Liang et al., 2018). This pattern was also described for SlDRP2A Arabidopsis orthologs: AtDRP2s, and for clathrin heavy and light chain proteins (Fujimoto et al., 2010; Huang et al., 2015). SlDRP2A partially colocalizes with the early endosome/*trans*-Golgi network (EE/TGN) marker, VHAa1 (Ivanov and Harrison, 2014), having a Pearson correlation coefficient of 0.45 ± 0.016 (Figure 2A and Supplementary Figure S3A). We further analyzed SlDRP2A localization using the late endosome/multivesicular body (LE/MVB) marker, FYVE (Figure 2A and Supplementary Figure S3A) (Vermeer et al., 2006; Simon et al., 2014) and the Golgi maker, Sialyltransferase protein (ST; Figure 2A and Supplementary Figure S3A) (Saint-Jore et al., 2002). Neither FYVE nor ST colocalize with SlDRP2A (Pearson correlation coefficient ≤0.2), indicating minimal presence on Golgi compartments and LE/MVB, as expected for a *bonafide* dynamin. Live cell imaging of *N. benthamiana* epidermal cells overexpressing SlDRP2A-mCherry and LeEIX2-GFP, demonstrates that the two proteins colocalize at the PM (Pearson correlation coefficient = 0.43 ± 0.040; Figure 2B and Supplementary Figure S3B) providing physical context for SlDRP2A-LeEIX2 protein interaction.

### SlDRP2A Enhances EIX Induced Defense Responses

Components of the endomembrane machinery, and DRPs in particular, have been extensively implicated as involved in defense responses and often act as targets to pathogen effectors (Toruno et al., 2016; Valencia et al., 2016). Therefore, following the identification of SlDRP2A as an LeEIX2 associating protein (Figure 1 and Supplementary Table S1), as well as the observation that the two proteins partially colocalize (Figure 2B), we examined a possible effect of SlDRP2A on EIX-mediated defense responses.

**FIGURE 3:**
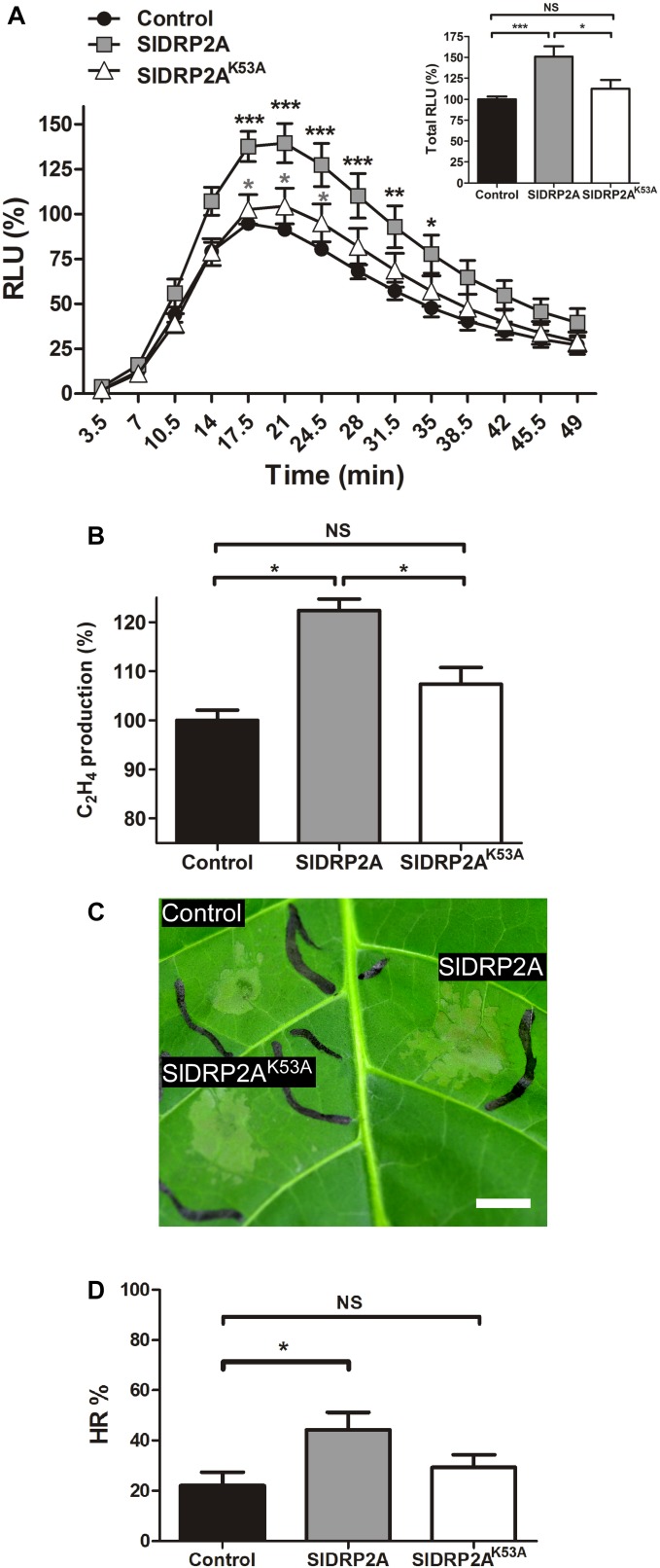
SlDRP2A enhances EIX-mediated immune responses. *N. tabacum* plants were transiently transformed with free mCherry (control), SlDRP2A-mCherry or SlDRP2A^K53A^-mCherry and elicited with EIX. **(A)** ROS oxidative burst after EIX elicitation was normalized to the control peak value in each experiment. Data of six independent replicates is represented as average ± SEM. Asterisks in black represent statistical significance between control and SlDRP2A and asterisks in gray represent statistical significance between control and SlDRP2A^K53A^ in two-way ANOVA and Bonferroni post-test (^*^p ≤ 0.05. ^∗∗^*p* ≤ 0.01, ^∗∗∗^*p* ≤ 0.001). No significant difference was observed between Control and SlDRP2A^K53A^. **(B)** Samples were treated with EIX or mock (water) for 4 h. Ethylene was measured using a gas-chromatograph. Ethylene production was defined as the ΔEthylene (Ethylene_+EIX_ – Ethylene_mock_), and control was defined as 100%. Data is represented as average ± SEM of five independent experiments. Asterisks represent statistical significance in one-way ANOVA and Tukey post-test (^*^p < 0.01, NS = no significant difference). **(C,D)** Hypersensitive Response (HR) development was determined by measuring the HR lesion area 24 h after EIX infiltration. HR% is defined as the percentage of the infiltrated area. Data is represented as average ± SEM of five independent replicates. Asterisks represent statistical significance in one-way ANOVA and Tukey post-test (^*^p ≤ 0.05; NS = no significant difference).

Overexpression of SlDRP2A leads to an increase in EIX-mediated ROS burst and ethylene production by 50 and 20%, respectively (Figures 3A,B). Additionally, SlDRP2A accelerates EIX-mediated HR, doubling the HR area as compared to HR developed in the control 24 h after EIX elicitation (Figures 3C,D). Overexpression of endocytic machinery components can affect trafficking and composition of cellular membranes (Moore and Murphy, 2009), alluding to the activity of the vesicles. To examine a possible effect of SlDRP2A on plant immunity without elicitation, in steady-state conditions, we conducted similar assays without elicitation, and no changes in ROS, ethylene production, or HR were observed (Supplementary Figures S4A–C).

To examine a possible functional role of SlDRP2A activity in mediation of LeEIX2 signaling, we generated a GTPase mutated form of SlDRP2A. We replaced lysine at position 53 with alanine (Figure 1 and Supplementary Figure S2), leading to a mutated SlDRP2A, termed SlDRP2A^K53A^. This mutation is predicted to cause loss of SlDRP2A function (Marks et al., 2001), and a similar mutation in Arabidopsis DRP2 was shown to produce a dominant negative effect (Taylor, 2011). Indeed, the GTPase-mutated form of SlDRP2A is not able to enhance EIX-mediated defense responses (Figures 3A–D). SlDRP2A^K53A^ does not affect ROS burst or ethylene production caused by EIX treatment (Figures 3A,B) and cannot accelerate the development of EIX-mediated HR (Figures 3C,D). These results indicate that the GTPase activity of SlDRP2A is essential for its regulatory role in LeEIX2-immune signaling and EIX-mediated defense. Similar expression levels were observed for SlDRP2A and SlDRP2A^K53A^ (Supplementary Figure S4D), and no changes in ROS, ethylene production, or HR were observed in steady-state conditions when overexpressing either forms of the protein (Supplementary Figures S4A–C).

### SlDRP2A Increases the Size and Density of LeEIX2 Endosomes but Not Flot1 Foci

**FIGURE 4:**
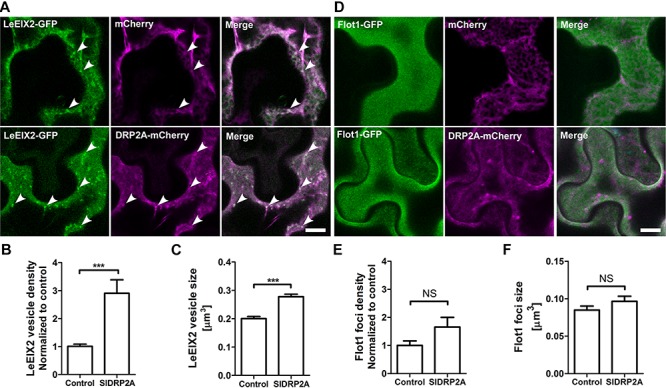
SlDRP2A alters LeEIX2, but not Flot1A sub-cellular localization. Confocal microscopy of *N. benthamiana* epidermal cells transiently expressing Flot1-GFP or LeEIX2-GFP and free-mCherry or SlDRP2A-mCherry. Number and size of foci/vesicles were determined using 3D Object counter function from FIJI-ImageJ. Representative images are shown, arrowheads indicate vesicles. Scale bar 10 μm. Data represented as mean ± SEM. *N* ≥ 25 images. **(A)** LeEIX2-GFP and free-mCherry or SlDRP2A-mCherry. **(B,C)** Quantification of LeEIX2-GFP foci number and size was made using 3D Object counter function from FIJI-ImageJ, average ± SEM is shown. **(D)** Flot1-GFP and free-mCherry or SlDRP2A-mCherry. **(E,F)** Quantification of Flot1-GFP vesicles number and size. Quantification of LeEIX2 vesicle and Flot1 foci number and size were made using 3D Object counter function from FIJI-ImageJ, average ± SEM is shown. Asterisks represent statistical significance in one-way ANOVA and Tukey post-test (^***^p ≤ 0.001).

LeEIX2-GFP is a transmembrane protein localized primarily at the PM, and its internalization is induced after EIX elicitation, in a clathrin dependent process (Sharfman et al., 2011). DRPs have a potential role in the process of LeEIX2 internalization. In order to test a possible role of SlDRP2A in membrane trafficking and particularly in CME, we utilized LeEIX2 and Flot1 which are known to be internalized via CME and clathrin independent endocytosis (CIE), respectively (Sharfman et al., 2011; Li et al., 2012). Both proteins were coexpressed with SlDRP2A in *N. benthamiana* and analyzed by live cell imaging. When SlDRP2A is overexpressed with LeEIX2, it leads to a 3-fold increase in LeEIX2 endosomal localization and augments the size of these endosomes (Figures 4A–C and Supplementary Figure S5A). However, overexpression of SlDRP2A does not affect Flot1 localization, size or density of Flot1 foci (Figures 4D–F and Supplementary Figure S5B). This differential effect of SlDRP2A suggests that SlDRP2A participates in CME, similarly to its Arabidopsis orthologs AtDRP2s (Fujimoto et al., 2010; Taylor, 2011).

### SlDRP2A Alters LeEIX2 Trafficking

We have previously reported that EIX treatment leads to an increase in LeEIX2 endosomal density (Bar and Avni, 2009; Sharfman et al., 2011). Moreover, we showed LeEIX2 signals from endosomal compartments, and that modifying LeEIX2 endosomal content can affect the intensity of its transmitted signal (Sharfman et al., 2011). Due to the significant role of CME in LeEIX2 signal transduction (Sharfman et al., 2011), and our results demonstrating SlDRP2A alters LeEIX2 endomembrane localization (Figures 4A–C), we examined a possible effect of SlDRP2A on EIX-triggered endocytosis of LeEIX2.

**FIGURE 5:**
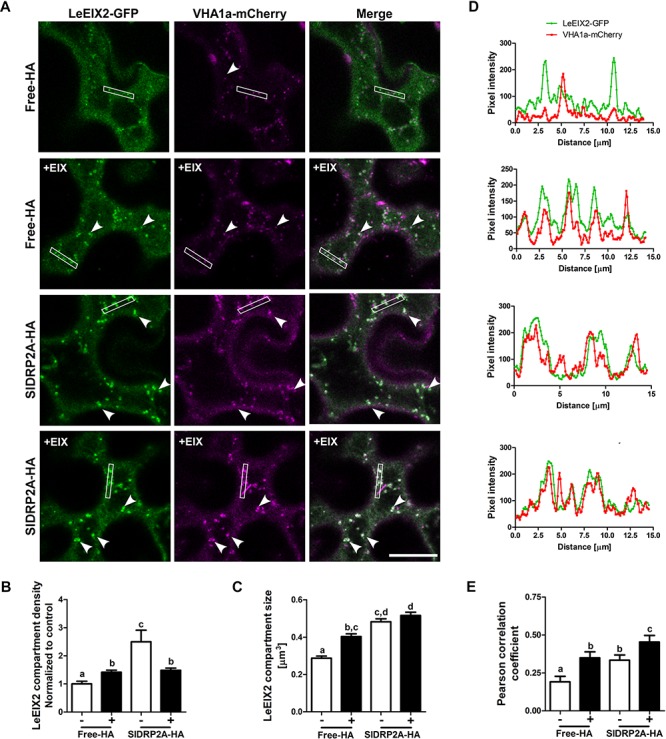
SlDRP2A increases LeEIX2 endocytosis and EE/TGN localization after EIX elicitation. Confocal microscopy of *N. benthamiana* epidermal cells transiently expressing LeEIX2-GFP, VHAa1-mCherry and free-HA or SlDRP2A-HA in steady state or after EIX elicitation. **(A)** Representative images of LeEIX2-GFP and VHAa1-mCherry co-expressed with free-HA or SlDRP2A-HA in steady state or after EIX elicitation. **(B,C)** LeEIX2 compartment density and size were determined using 3D Object counter function from FIJI-ImageJ. **(D)** Intensity profile of the white rectangle section in **(A)** was determined using the plot profile function from FIJI-ImageJ. **(E)** Co-localization between LeEIX2-GFP and VHAa1-mCherry was analyzed using Coloc2 from FIJI-ImageJ and Pearson correlation coefficient is shown. Scale bar 20 μm. Data are presented as mean ± SEM. *N* ≥ 30 images. Arrowheads indicate LeEIX2 compartments. Statistical significance was evaluated by two-way ANOVA and Bonferroni post-test (*p* ≤ 0.05).

When transiently coexpressing LeEIX2-GFP and free HA (control) in *N. benthamiana*, we observed an increase in size and density of LeEIX2 endosomes upon EIX treatment (Figures 5A–C). The increase in LeEIX2 endosomal content is transient, peaking after 30 min and subsiding after 60–90 min (Bar and Avni, 2009), and can be interpreted as RME/cargo internalization from the apoplast. The increase in LeEIX2 endocytic vesicles upon EIX treatment replicates our previous results (Leibman-Markus et al., 2018). Following EIX elicitation, intracellular localization and colocalization of LeEIX2 with VHAa1, an EE/TGN marker, increased (Figures 5A–C, Free-HA samples, Supplementary Figure S6), confirming that ligand induced internalization of LeEIX2 drives LeEIX2 into EE/TGN compartments, where signaling is likely taking place (Bar and Avni, 2009; Sharfman et al., 2011). To rule out an effect of SlDRP2A on LeEIX2 amounts within the cell, due to an effect on protein expression or stability, we conducted western blotting analysis on LeEIX2 when expressed together with free-mCherry or SlDRP2A-mCherry (Supplementary Figure S7), demonstrating that LeEIX2 protein amounts do not increase when co-expressed with SlDRP2A. We further assessed 25 images of four experiments under identical microscopy conditions, demonstrating that SlDRP2A causes a decrease in total pixel intensity of LeEIX2-GFP (Supplementary Figure S7A), supporting the notion that the increase in LeEIX2 mediated signaling occurs via a change in protein internalization rather than levels.

In order to assess the effect of SlDRP2A on LeEIX2 trafficking, LeEIX2-GFP, VHAa1-mCherry and SlDRP2A-HA or free-HA (control), were coexpressed in *N. benthamiana* epidermal cells and live cell imaging was performed. When SlDRP2A is overexpressed, LeEIX2-endosome density and size increase in steady-state conditions (Figures 5A–C and Supplementary Figure S6). LeEIX2 colocalization with the VHAa1 EE/TGN marker in a SlDRP2A overexpression background is higher than the colocalization in the control (Figure 5E, EIX-untreated samples). Interestingly, we observed a larger proportion of LeEIX2 on EE/TGN following EIX elicitation in a SlDRP2A overexpression background (Figure 5E). This suggests that both SlDRP2A overexpression and EIX elicitation act on the same sub-population of VHAa1 positive EE/TGN. Though SlDRP2A increases LeEIX2 endosomal localization prior to EIX treatment (Figures 4A–C, 5A–C), this in itself does not lead to an increase in plant defense responses (Supplementary Figures S4A–D), which are only activated after EIX exposure. Thus, though LeEIX2 signals from endosomes (Sharfman et al., 2011), entry into EE/TGN is not sufficient to activate the receptor, as defense signaling requires ligand binding, as we previously reported (Leibman-Markus et al., 2018).

### SlDRP2A Increases FLS2 Mediated Defense and Endosomal Localization

**FIGURE 6:**
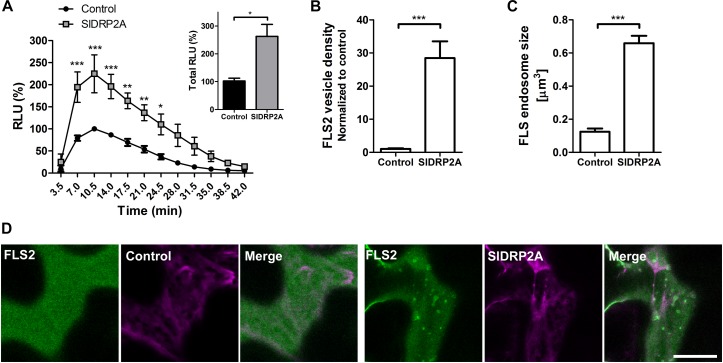
SlDRP2A increases FLS2 mediated defense and endosomal localization. **(A)** ROS oxidative burst after flg22 elicitation was normalized to the control peak value in each experiment. Data of four independent replicates is represented as average ± SEM. Asterisks represent statistical significance in two-way ANOVA and Bonferroni post-test (^*^p ≤ 0.05, ^∗∗^*p* ≤ 0.01, ^∗∗∗^*p* ≤ 0.001). **(B–D)** Confocal microscopy of *N. benthamiana* epidermal cells transiently expressing FLS2-GFP and free-mCherry or SlDRP2A-mCherry. **(B,C)** Number and size of foci/vesicles were determined using 3D Object counter function from FIJI-ImageJ. Data represented as mean ± SEM. *N* ≥ 18 images. **(D)** Representative images are shown, arrowheads indicate vesicles. Scale bar 20 μm.

We examined the possibility of SlDRP2A participating in signal transduction of additional PRR proteins by testing a possible role of SlDRP2A in FLS2-mediated defense. We measured the ROS burst after flg22 elicitation when transiently expressing DRP2A in *N. benthamiana*. SlDRP2A enhanced the response more than 2.2 fold (Figure 6A and Supplementary Figure S8). No response was detected without flg22 elicitation. Our results contradict those observed in *A. thaliana* and *N. benthamiana*, where it was found that overexpression of DRP2 suppressed flg22 induced ROS burst. This could potentially indicate that SlDRP2A is not a true ortholog of the *A. thaliana* and *N. benthamiana* proteins used in those works. An alternative explanation to this contradiction, could relate to species-specific effects, although, interestingly, in the case of *N. benthamiana* and consistently with our results, a reduction in flg22-induced ROS burst upon NbDRP2 silencing was also observed in 40% of experiments (Chaparro-Garcia et al., 2015). To examine whether the underlying mechanism is similar to that of the interaction with LeEIX2 and correlate our results with those previously reported for DRP2 in *A. thaliana* and *N. benthamiana*, we examined the effect of SlDRP2A overexpression on FLS2 endosomes. SlDRP2A significantly increases FLS2 vesicle density (Figures 6B,D) and size (Figures 6C,D) as compared to control, indicating the increase in defense signaling is likely based on a similar mechanism as with LeEIX2. Interestingly, here, our results correlate with those of Chaparro-Garcia et al. (2015), who found that NbDRP2 silencing caused a significant reduction in the number of FLS2 endosomes. As was done with LeEIX2, to rule out an effect of SlDRP2A on FLS2 amounts within the cell, due to an effect on protein expression or stability, we assessed 18 images of three experiments under identical microscopy conditions, demonstrating that SlDRP2A causes a decrease in total pixel intensity of FLS2-GFP, making an increase in protein expression or stability highly unlikely (Supplementary Figure S7B). Thus, supporting the notion that the increase in FLS2-mediated signaling occurs via a change in protein internalization, in accordance with previous results which found that DRP2 is required for FLS2 internalization in *N. benthamiana* (Chaparro-Garcia et al., 2015).

Taken together, our results indicate that SlDRP2A is a component of the endocytic machinery, regulating PRR internalization and signal propagation.

## Discussion

Plant immune responses and pathogen virulence both rely on trafficking within the plant cell. Plants use trafficking mechanisms to regulate both steady-state events, such as receptor and membrane homeostasis, and acute events, such as response to pathogen exposure. We have previously demonstrated that endocytosis is crucial for LeEIX2-defense signaling (Ron and Avni, 2004; Bar and Avni, 2009; Sharfman et al., 2011), and here, we report the involvement of a dynamin-related endocytic machinery component, SlDRP2A, acting as a positive regulator of LeEIX2 receptor trafficking and LeEIX2-mediated defense responses.

Previous works have highlighted the importance of endocytic events in plant immunity (Robatzek et al., 2006; Bar et al., 2009; Sharfman et al., 2011; Mbengue et al., 2016; Postma et al., 2016). RME, including elicitor/cargo internalization, has been described in several cases (Ron and Avni, 2004; Bar and Avni, 2009; Sharfman et al., 2011; Mbengue et al., 2016; Ortiz-Morea et al., 2016). In addition to the importance of cargo internalization to the process of RME in immune signaling, we have previously shown that EIX-immune signaling is partially dependent on events occurring directly upon endosomes (Sharfman et al., 2011). In line with the concept of pivotal roles for endocytic machinery components in signaling events, dynamins have previously been shown to play a part in pathogen resistance (Tang et al., 2006; Viotti et al., 2010; Smith et al., 2014; Chaparro-Garcia et al., 2015). DRP2 has been implicated in plant immunity as a target of immune suppression by the *Phytophtora infestans* effector AVR3a (Chaparro-Garcia et al., 2015). A previous work by Smith et al. (2014) reported an *Atdrp2b* mutant displaying reduced induction of PR1 gene expression after flg22 elicitation, and increased susceptibility to *Pseudomonas syringae*, in line with our results. Our results suggest that DRP2 is involved in FLS2 endocytosis and defense signaling, positively regulating plant defense via endocytosis and trafficking mechanisms (Smith et al., 2014; Chaparro-Garcia et al., 2015). Additionally, it is also possible that DRP2 may affect PRR recycling rates for both LeEIX2 and FLS2, causing more receptor to be present in signaling compartments, resulting in an increase in signaling events.

Here, we provide the first report of the involvement of a tomato DRP in immune signaling. SlDRP2A enhances LeEIX2 and FLS2 RME and EIX and flg22 induced immune-responses (Figures 3–5). This points to a possible general role for DRPs in the control of signaling events originating from membranal vesicles, as we and others suggested for dynamins in the past (Bar and Avni, 2009; Sharfman et al., 2011; Smith et al., 2014; Chaparro-Garcia et al., 2015). Interestingly, silencing DRP2 in *N. benthamiana* reduced flg22-induced FLS2 internalization (Chaparro-Garcia et al., 2015), and mutating Arabidopsis DRP enhanced disease resistance 3 (DRP1E), led to increased susceptibility against *Botrytis cinerea* (Tang et al., 2006). The enhancing effect of SlDRP2A overexpression on EIX and flg22 triggered oxidative burst is opposite to that observed for AtDRP2B and *N. tabacum* DRP2, on flg22-triggerd oxidative burst, where a negative regulation was reported (Smith et al., 2014; Chaparro-Garcia et al., 2015). Although in the case of *N. benthamiana*, consistently, with our results, a reduction in flg22 induced oxidative burst upon NbDRP2 silencing was also observed in 40% of experiments (Chaparro-Garcia et al., 2015). Oxidative burst is produced mainly due the activity of RBOH enzymes, which are PM proteins that could also be endocytic targets (Hao et al., 2014). This observed difference could relate to differences in function between the tested DRP proteins and/or alterations in immune system properties in different plant species.

SlDRP2A enhances LeEIX2 and FLS2 endosome size and density in steady-state conditions, but does not affect immune responses prior to EIX elicitation. These results are in line with our previous reports that elicitor recognition is required for defense response activation. The increase in LeEIX2 endosomal localization in steady-state conditions was previously observed when overexpressing SlNRC4a, an NLR protein (Leibman-Markus et al., 2018). Like in the case of SlDRP2A, SlNRC4a led to an elicitor-dependent enhancement of immune responses (Leibman-Markus et al., 2018). Taken together, and given our previous work that LeEIX2 signaling occurs from endosomes, these results point to a possible priming effect occurring through EE/TGN.

SlDRP2A has a PH domain, which is thought to contribute to membrane curving and breakdown of the lipid bilayer through its binding to acidic phospholipids and phosphatidyl inositol-4,5-bisphosphate (PI4,5P2) on donor membranes (Schmid and Frolov, 2011). SlDRP2A also possesses a C-terminal PRM harboring a conserved PXXP amino acid motif, which can interact with SH3 domain-containing proteins to position dynamin at vesicle formation sites (Fujimoto and Tsutsumi, 2014). These structural analyses could speak to the dual role of SlDRP2A-structurally enabling receptor internalization upon ligand binding, while at the same time bringing associating partners required for signal propagation into close proximity of the receptors. Mutating the SlDRP2A GTPase domain abolishes the enhancement of signal propagation (Figure 3).

Our previous work has demonstrated that LeEIX2-mediated signaling can occur directly at endosomal locales, probably EE or TGN (Bar and Avni, 2009; Sharfman et al., 2011). It is therefore not surprising that an increase in EE/TGN content generates an increase in LeEIX2-mediated signaling. However, given that intact GTPase activity of SlDRP2A is required for enhancing EIX-induced signals, it is tempting to speculate that SlDRP2A is involved in the signaling events themselves, possibly by bringing together interacting partners required for signal propagation. Endogenous control of expression levels of SlDRP2A could potentially serve as a cellular mechanism to control endomembrane content, with SlDRP2A quantities acting as a bottle-neck for membranal vesicle formation and the meeting of partners required for signal propagation, ultimately controlling both the output and intensity of the transmitted signal. Data obtained from systems in which SlDRP2A and/or its interacting PRRs are expressed from their endogenous promoters and/or in stable transgenic plants will be valuable for further investigating this issue, should they be feasible.

Recently, AtDRP2A and AtDRP2B have been related to Arabidopsis/Turnip mosaic virus (TuMV) infection, where these DRPs likely participate in TuMV internalization, movement and replication favoring TuMV infection (Wu et al., 2018). This antagonistic dual role of DRPs in defense is an important trade-off to consider for plant breeding. Further analyses are required to elucidate the exact roles of DRPs in cellular signaling events and uncover the specific roles of different SlDRP2A domains in both structural and signaling contexts.

## Accession Numbers

SlDRP2A, Solyc11g03950; SlDRP2B, Solyc01g103130 and Solyc01g103120 (Genbank: XP_004230421); LeEIX2; Solyc07g008630; FLS2, AT5G46330.

## Data Availability

All datasets generated for this study are included in the manuscript and/or the Supplementary Files.

## Author Contributions

AA, MB, ML-M, and LP conceived and designed the study. AA and MB secured funding for the project. LP, ML-M, and SS formulated the methodology and carried out the experiments. ML-M, LP, AA, and MB analyzed the data. All authors contributed to the writing of the manuscript.

## Conflict of Interest Statement

The authors declare that the research was conducted in the absence of any commercial or financial relationships that could be construed as a potential conflict of interest.

## Footnotes

1 ftp://ftp.solgenomics.net/proteomics/Solanum_lycopersicum/

2 https://www.ncbi.nlm.nih.gov/Structure/cdd/wrpsb.cgi
