# Supplementary figures and images for: Tomato Dynamin Related Protein 2A Associates With LeEIX2 and Enhances PRR Mediated Defense by Modulating Receptor Trafficking
Source: Front Plant Sci. 2019 Jul 19;10:936. doi: 10.3389/fpls.2019.00936 (PMC6658876; doi:10.3389/fpls.2019.00936)

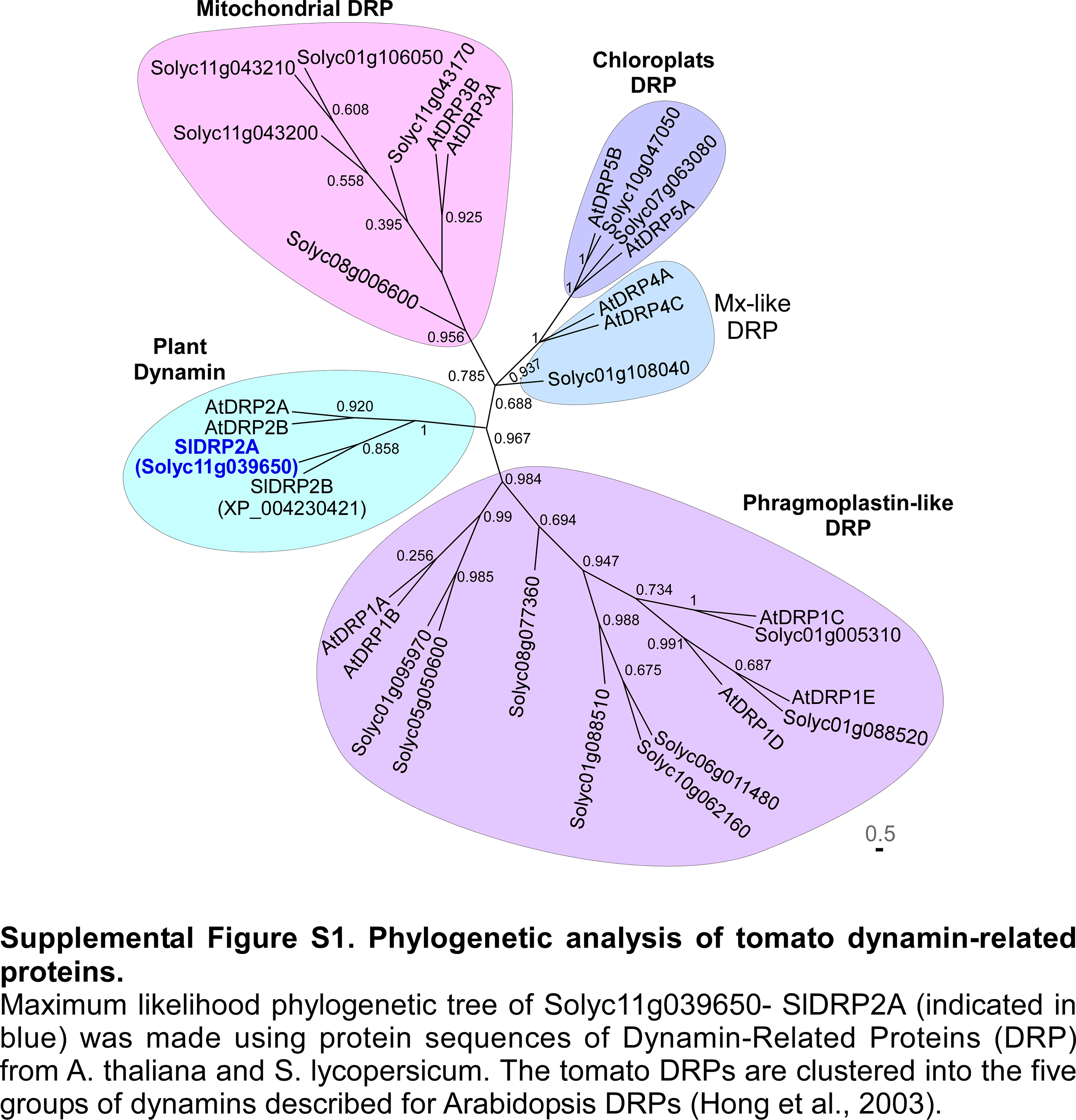

Supplement: Supplementary file 1 [file Image_1.TIF]

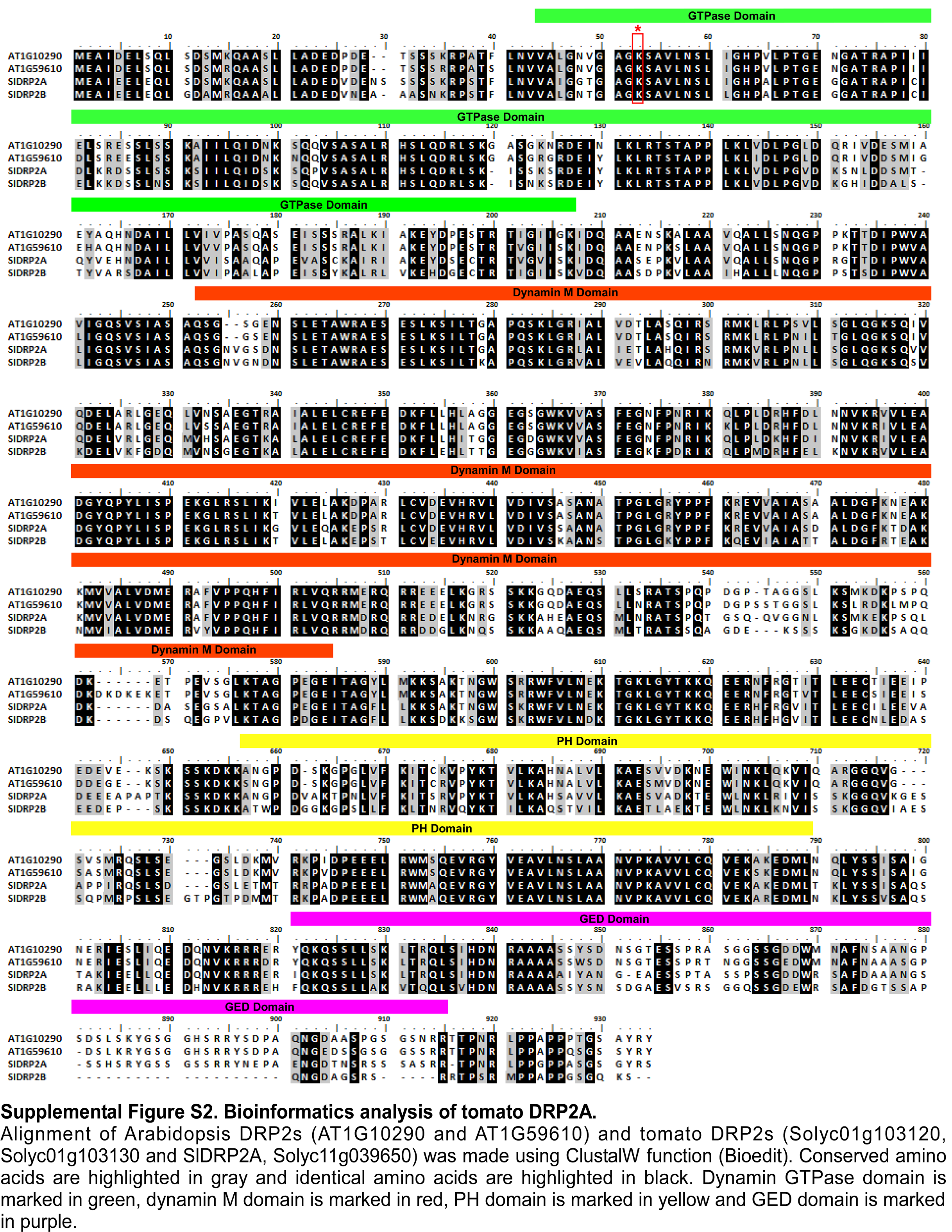

Supplement: Supplementary file 2 [file Image_2.TIF]

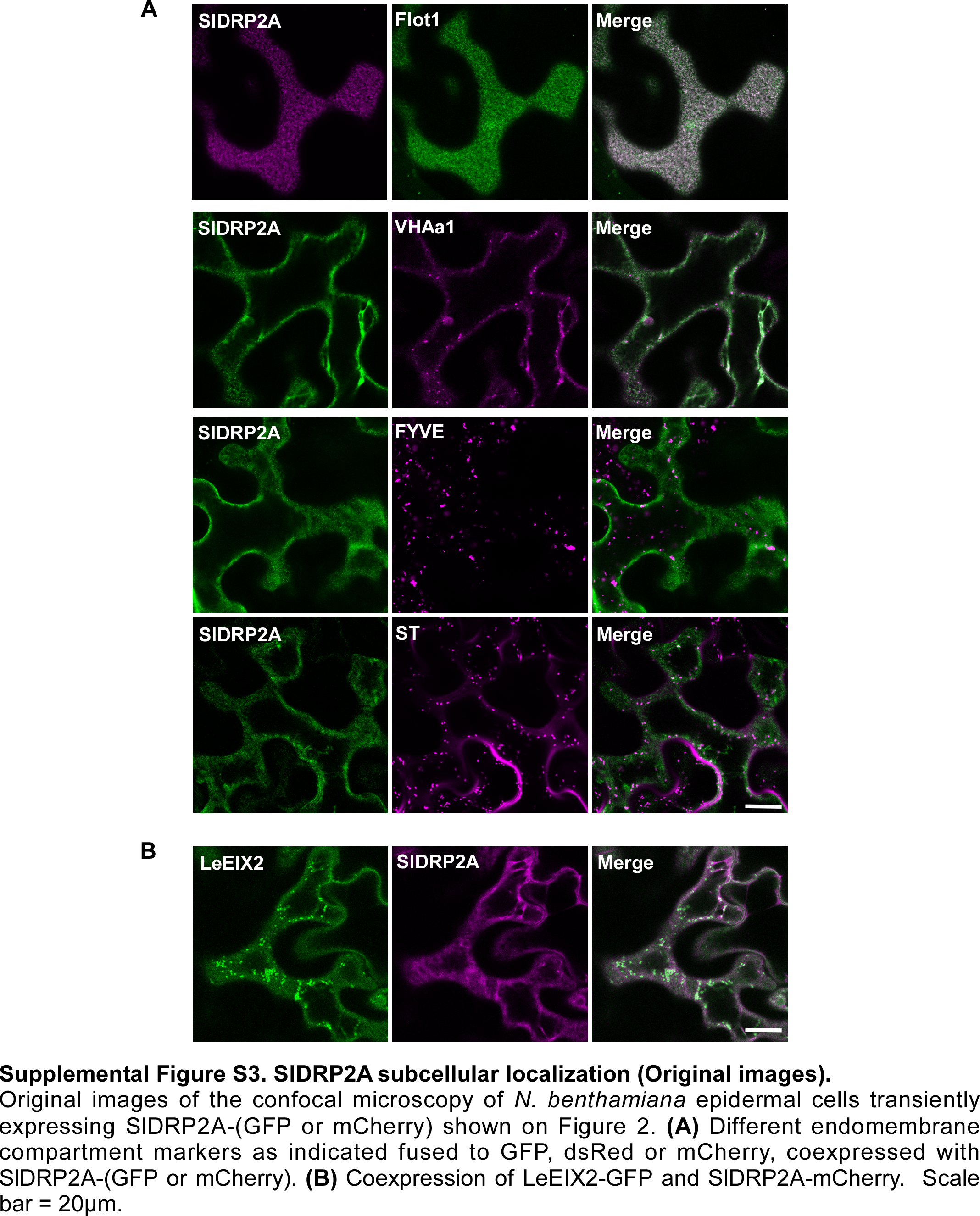

Supplement: Supplementary file 3 [file Image_3.TIF]

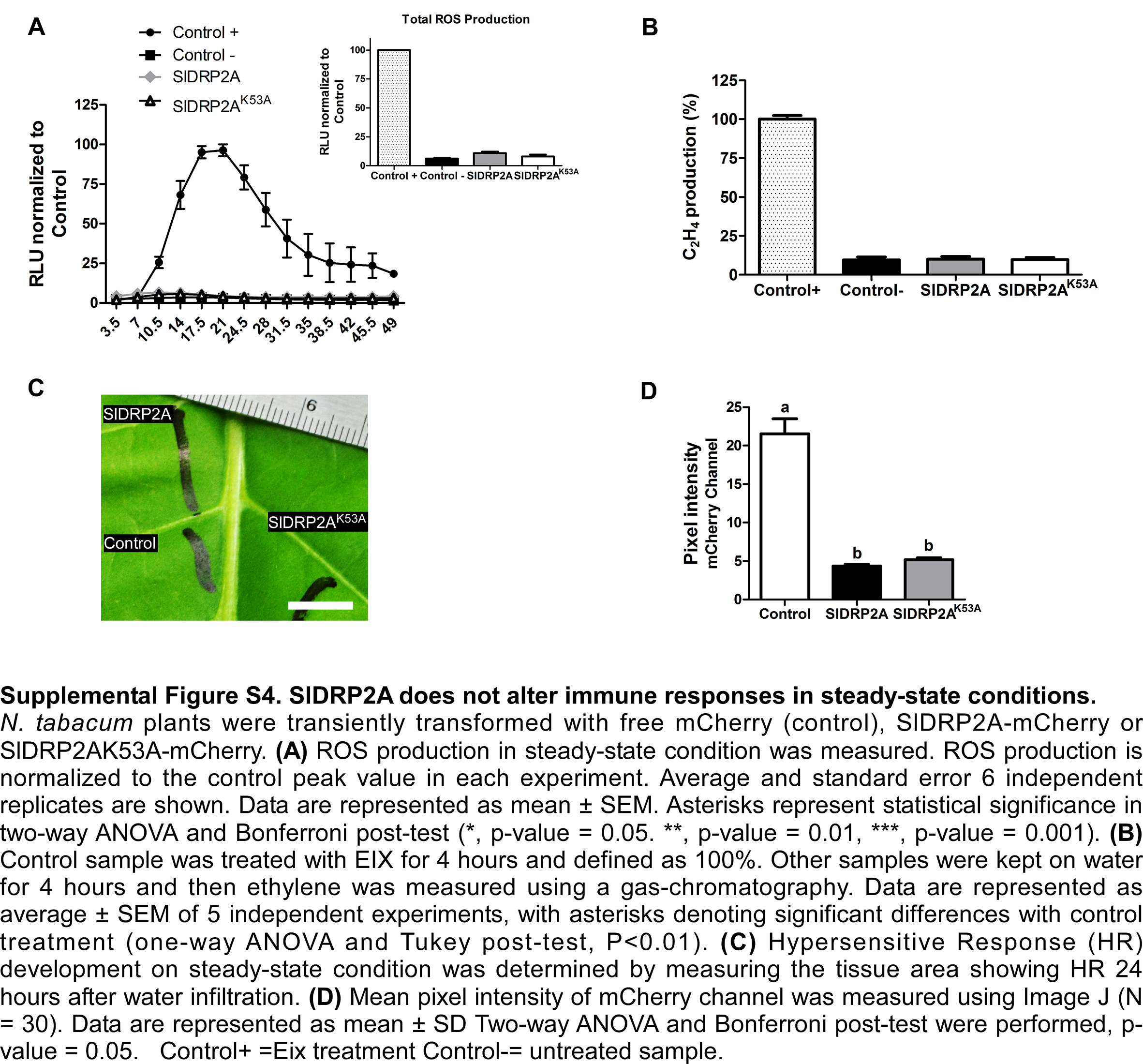

Supplement: Supplementary file 4 [file Image_4.TIF]

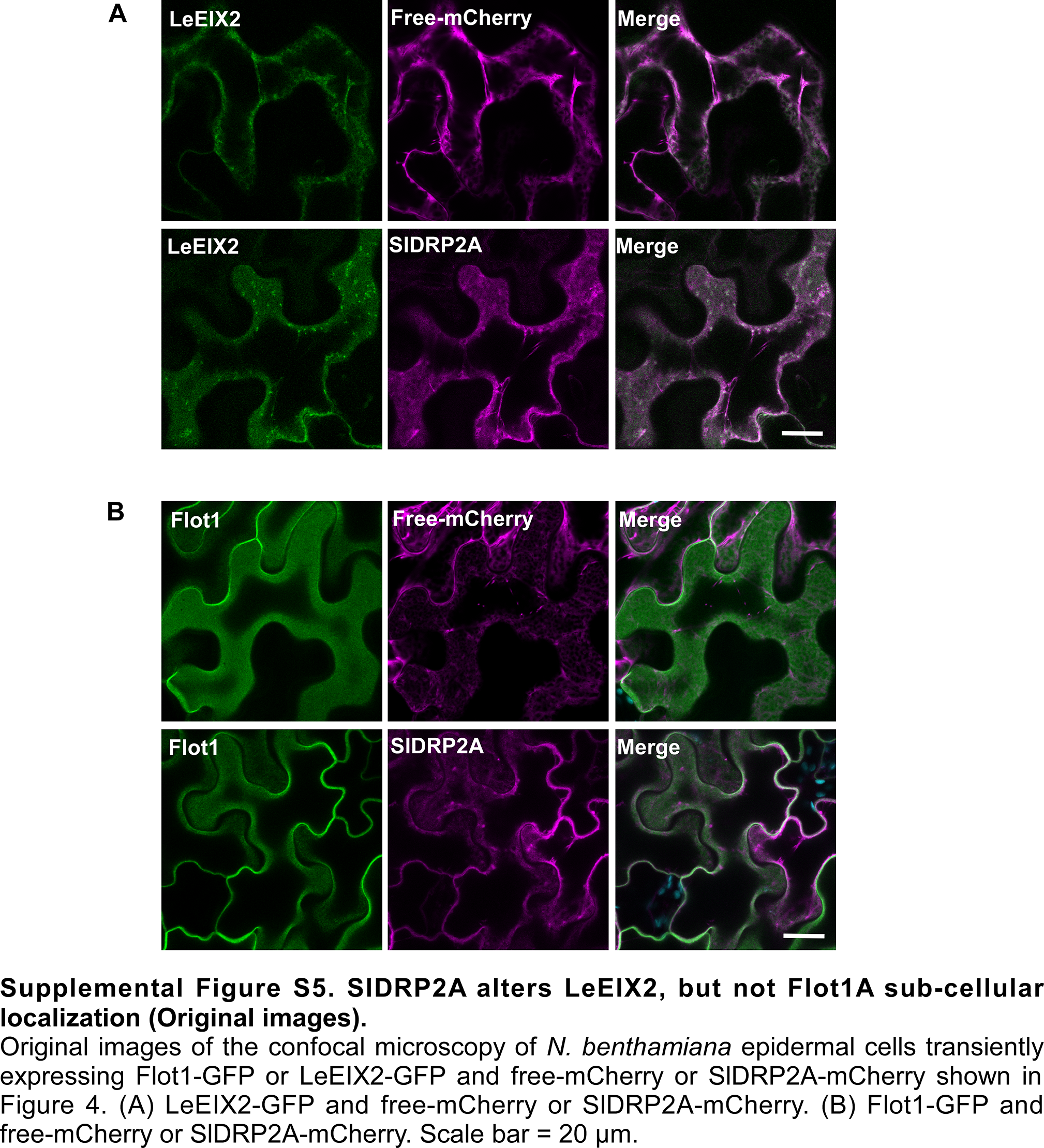

Supplement: Supplementary file 5 [file Image_5.TIF]

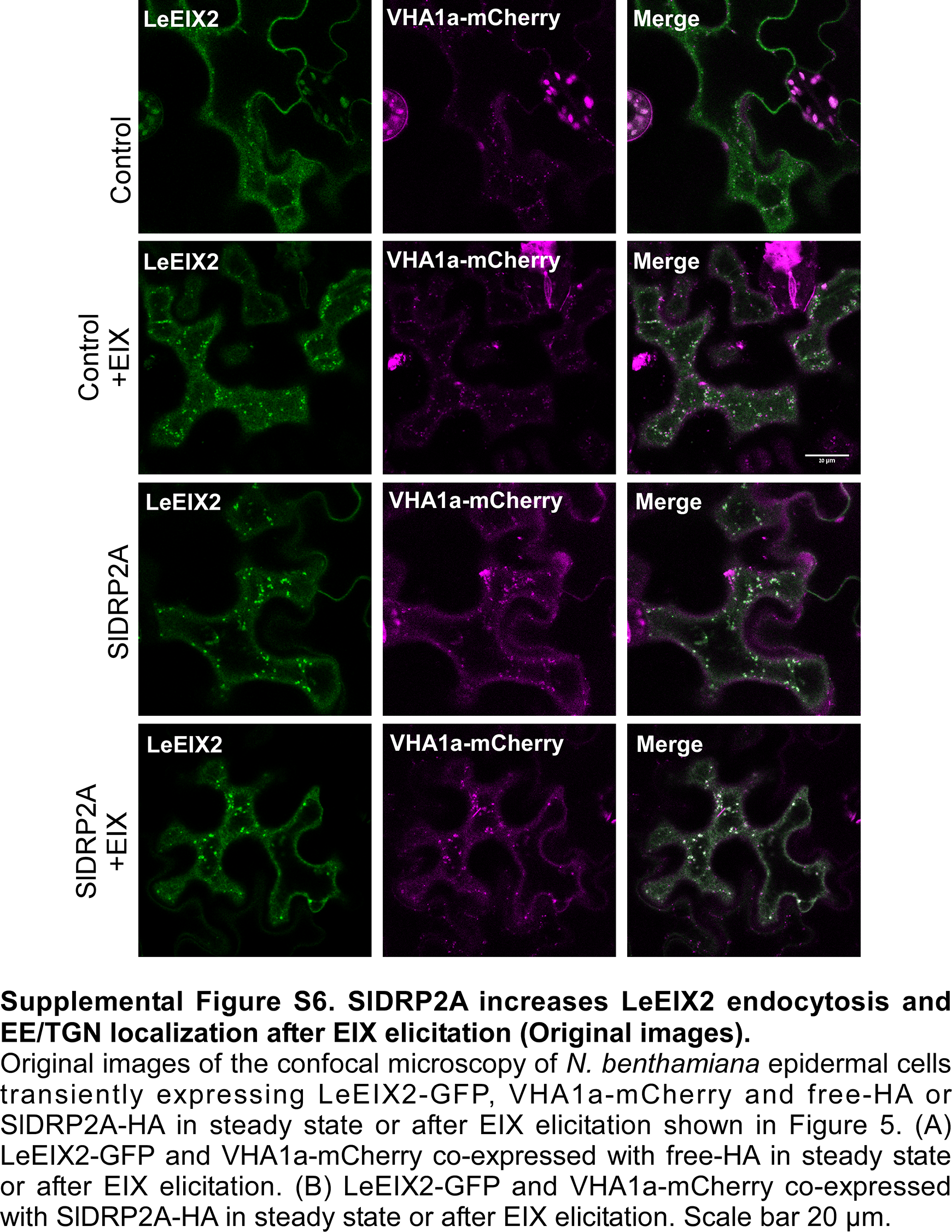

Supplement: Supplementary file 6 [file Image_6.TIF]

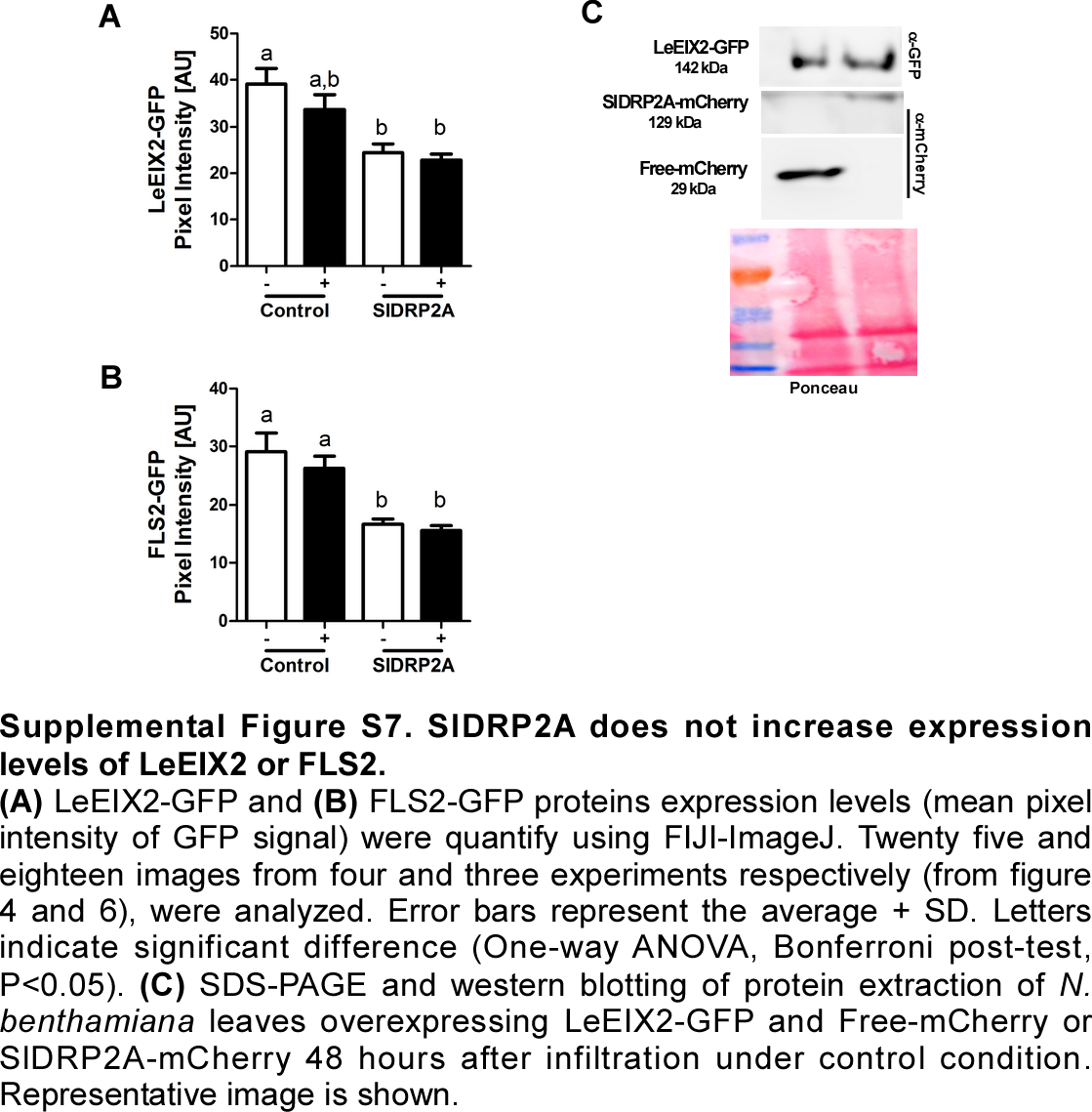

Supplement: Supplementary file 7 [file Image_7.tif]

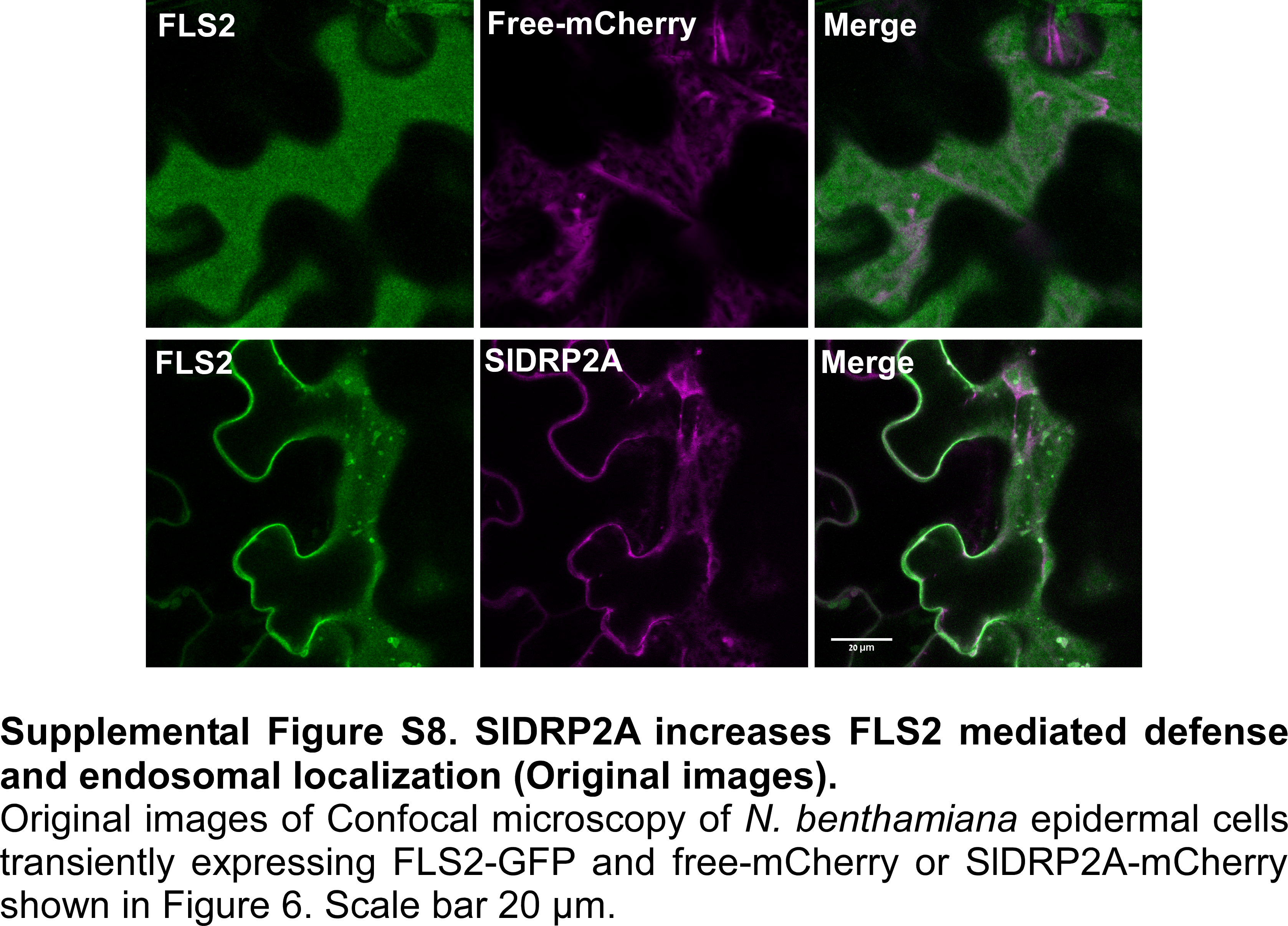

Supplement: Supplementary file 8 [file Image_8.TIF]
