# Supplementary material for: Tomato Dynamin Related Protein 2A Associates With LeEIX2 and Enhances PRR Mediated Defense by Modulating Receptor Trafficking
Source: Front Plant Sci. 2019 Jul 19;10:936. doi: 10.3389/fpls.2019.00936 (PMC6658876; doi:10.3389/fpls.2019.00936)
Supplement: Supplementary file 9 [file Table_1.DOCX]

**Table S1** Sequences and peptide identification probability (Prob) of peptides specifically matching predicted protein SlDRP2A from TL4 LeEIX2-GFP immunopurified samples.

| Protein name | **Accession number** | **Peptide sequence** | **Prob** | | **X! Tandem –log(e) score** |
| --- | --- | --- | --- | --- | --- |
| SlDRP2A | Solyc11g039650 | (K)IDQAASEPK(V) | | 99% | 1.69897 |
| SlDRP2A | Solyc11g039650 | (K)mVVALVDmER(A) | | 100% | 4.39794 |
| SlDRP2A | Solyc11g039650 | (K)SILTGAPQSK(L) | | 98% | 2.080922 |
| SlDRP2A | Solyc11g039650 | (K)SQPVSASALR(H) | | 100% | 5.113509 |
| SlDRP2A | Solyc11g039650 | (K)SQVVQDELVR(L) | | 100% | 2.148742 |
| SlDRP2A | Solyc11g039650 | (K)VLAAVQALLSNQGPR(G) | | 100% | 9.721247 |
| SlDRP2A | Solyc11g039650 | (K)VVASFEGNFPNR(I) | | 100% | 4.853872 |
| SlDRP2A | Solyc11g039650 | (R)ATSPQTGSQQVGGNLK(S) | | 100% | 4.387216 |
| SlDRP2A | Solyc11g039650 | (R)EVVAIASDALDGFK(T) | | 100% | 3.920819 |
| SlDRP2A | Solyc11g039650 | (R)EVVAIASDALDGFKTDAK(K) | | 100% | 7.040958 |
| SlDRP2A | Solyc11g039650 | (R)GYVEAVLNSLAANVPK(A) | | 100% | 9.468521 |
| SlDRP2A | Solyc11g039650 | (R)IVLEADGYQPYLISPEK(G) | | 100% | 8.431798 |
| SlDRP2A | Solyc11g039650 | (R)LPNLLSGLQGK(S) | | 100% | 3.420216 |
| SlDRP2A | Solyc11g039650 | (R)VLVDIVSSAANATPGLGR(Y) | | 100% | 10.20066 |

Deamidated asparagine denoted as “n” and oxidized methionine denoted as “m”.
